# Calcific tendonitis of the right epicondyle of the elbow: A case report

**DOI:** 10.1097/MD.0000000000041604

**Published:** 2025-03-21

**Authors:** Emmanuel Ackah, Deting Xue, Zhijun Pan

**Affiliations:** a Zhejiang University School of Medicine, Hangzhou, Zhejiang, China.

**Keywords:** calcified tendonitis, dystrophic calcification, elbow, extensor tendon, lateral epicondylitis

## Abstract

**Rationale:**

Calcific tendinitis frequently occurs in the shoulder, and while it may also occur in other joints, such as the hip, knee, wrist, and finger joints, its occurrence in the elbow joint is relatively rare. Therefore, it is susceptible to delayed diagnosis or misdiagnosis at the initial occurrence. Appropriate imaging and treatment should be considered immediately when suspecting calcific tendonitis.

**Patient concerns:**

We detailed a young female who presented with progressive right elbow pain for the past 3 years with no past injury to the elbow. She had undergone conservative management for 2 years but was unsuccessful. Her pain became more intense 1 week prior, to the extent that she could not use her right upper limb to perform daily activities, and her sleep was severely disturbed. Therefore, she needed a possible therapeutic relief.

**Diagnosis:**

Clinical findings included elbow stiffness and tenderness of the lateral epicondyle of the humerus, and Cozen assessment was positive. Computed tomography showed a high-density macular focus and smooth edge of the lateral condyle of the right humerus and dorsal side of the ulnar olecranon. The plain radiograph showed patches in the right lateral condyle of the humerus and the dorsal part of the olecranon, indicating calcification of common extensor tendinosis.

**Interventions:**

Initially, conventional care was given to the patient with analgesics, physical therapy, or a resting regimen to decrease the pain and reduce the tedious load on the extensor tendon. Measures such as pain score, range of motion, and follow-up imaging after 8 weeks began until 2 years. However, her pain did not improve. Therefore, she was advised for surgical therapy and subsequently underwent surgical exploration of the elbow under general anesthesia. Histopathological examination of the excised tissue revealed fibrous ligament tissue with calcifications.

**Outcomes:**

At the 6-month follow-up, the postoperative plain radiograph showed complete removal of the heterotopic bony growth from the lateral epicondylar area with no recurrence of the heterotopic bone formation around the elbow joint. She had no pain with full elbow function and a full range of elbow extension and flexion (visual analog scale score of 0/10).

**Lessons:**

Calcific tendonitis of the elbow is uncommon; hence, its diagnosis and treatment may be late due to its scarcity. Therefore, appropriate imaging and treatment should be considered immediately when calcified tendonitis is suspected. A literature review is also necessary since it is not a daily condition.

## 1. Introduction

Calcified tendinopathy or calcific tendonitis is a chronic condition in which calcium phosphate crystal deposits accumulate in the midsubstance of tendon fibers.^[1]^ Epidemiological studies have reported that the prevalence of calcified tendonitis is highly variable (3%–22% in the general population) depending on the location within the body.^[2]^ Calcific tendinitis frequently occurs in the shoulder, with an incidence of about 2.7% in the adult population.^[3]^ While it may also occur in other joints, such as the hip, knee, wrist, and finger joints, its occurrence in the elbow joint is relatively rare. Only a few cases of calcific tendonitis occurred in the elbow.^[4]^ Owing to its uncommonness, it is susceptible to delayed diagnosis or misdiagnosis at the initial occurrence. The treatment of calcific tendonitis is usually conservative, including nonsteroidal anti-inflammatory drugs, elbow support, immobilization of the elbow, physical therapy, and corticosteroid injection. Surgical treatment is considered when conservative therapy is unsuccessful. Calcific tendonitis of the elbow is a rare condition that many orthopedic surgeons are unaware of. Therefore, it is essential to make an accurate diagnosis and management plan. Women are affected slightly more often than men, with patients typically between 30 and 60 years of age.^[5]^ We present a case of a young adult female with calcified tendonitis.

## 2. Case presentation

**Figure 1.**
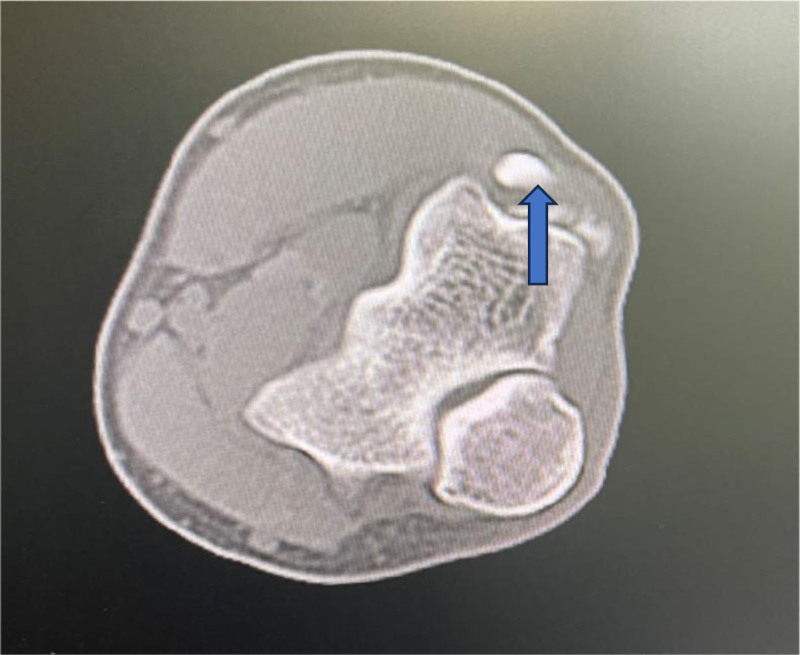
Computed tomography of high-density macular focus and smooth edge of the lateral condyle of the right humerus and dorsal side of the ulnar olecranon.

**Figure 2.**
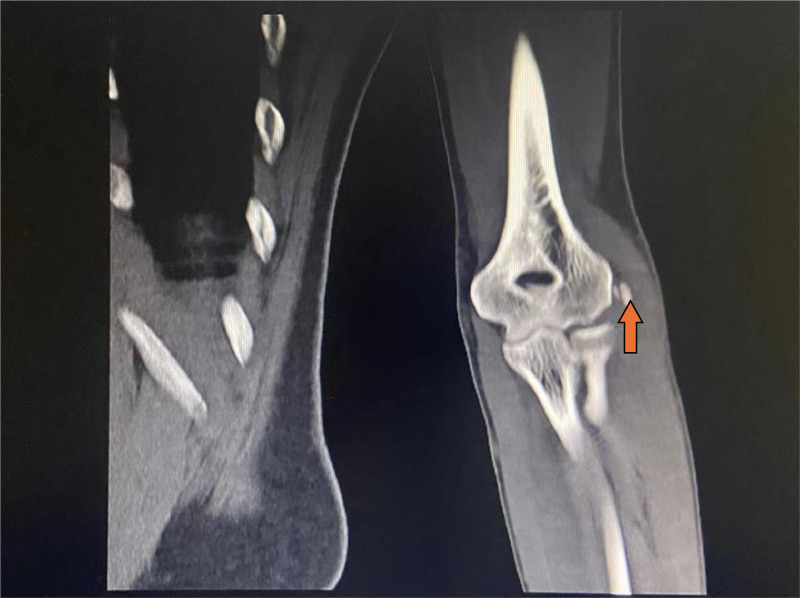
A radiograph of the right-hand shows calcification of the lateral epicondyle.

**Figure 3.**
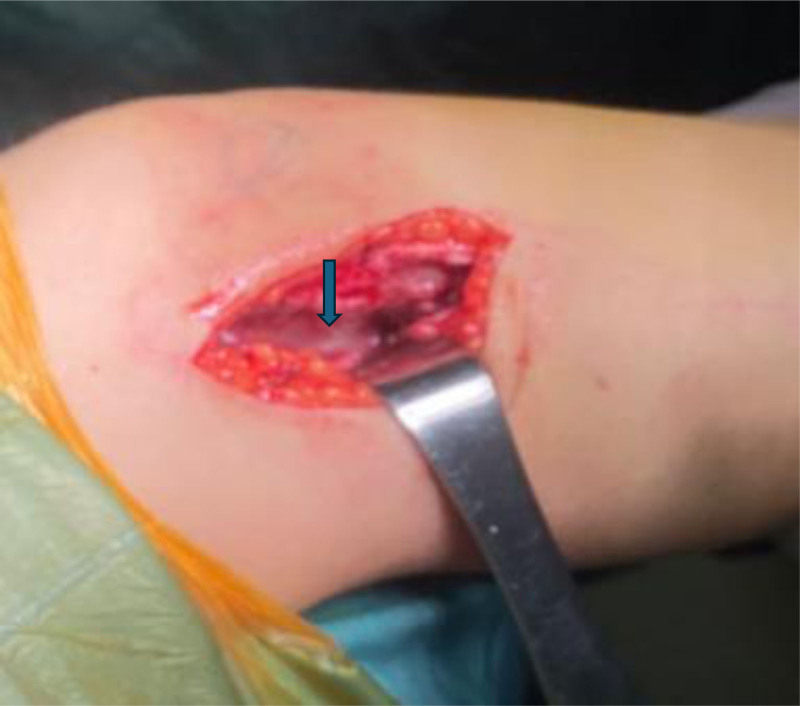
Shows a milky white liquid outflow from the common extensor tendon region with dystrophic calcification.

**Figure 4.**
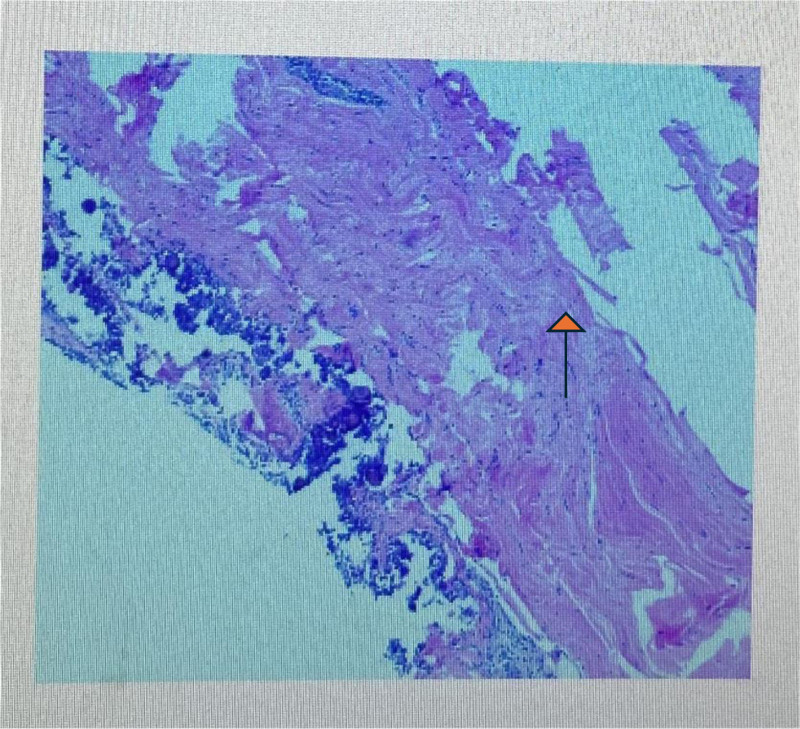
Histopathology showing revealed fibrous ligament tissue with calcifications (arrow).

**Figure 5.**
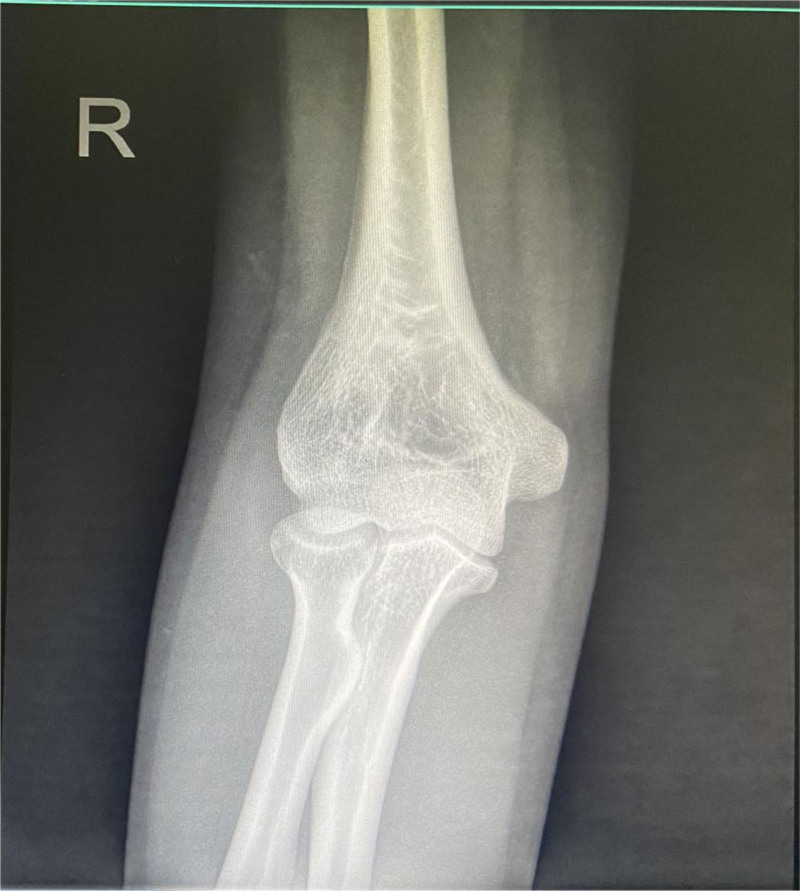
The postoperative plain radiograph showed a complete abolition of the heterotopic bony growth from the lateral epicondylar region.

**Figure 6.**
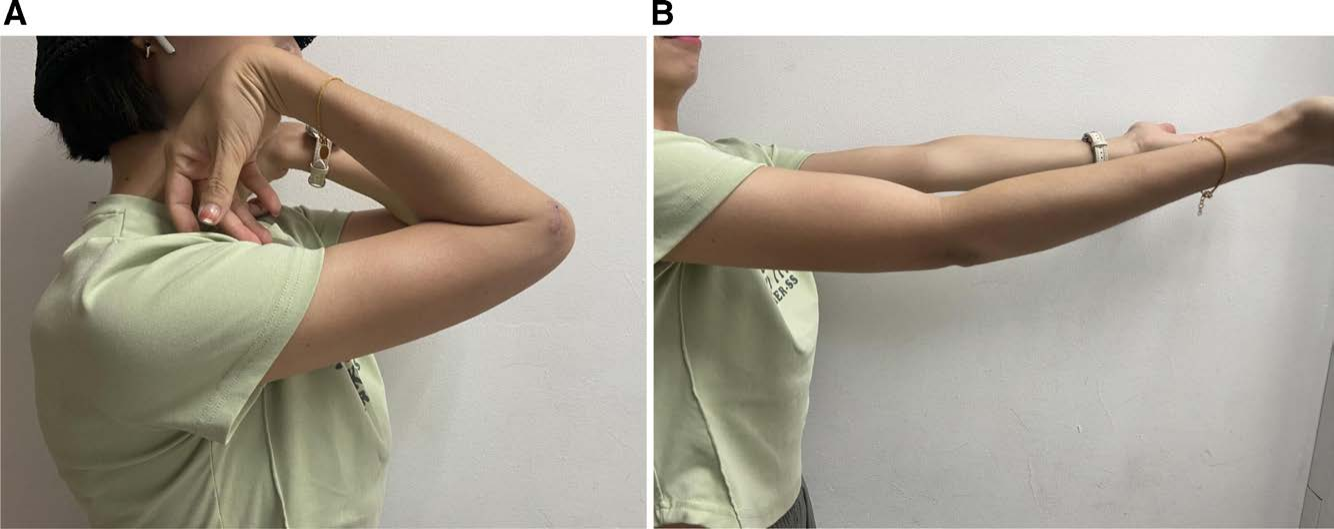
A follow-up examination after 6 months showed full extension and flexion of the elbow.

A 31-year-old female presented with right elbow pain for the past 3 years with no past injury to the elbow. Physical examination revealed elbow stiffness and tenderness of the lateral epicondyle of the humerus. Cozen assessment was positive, indicating a clinical diagnosis of lateral epicondylitis of the elbow. The plain radiograph showed patches in the right lateral condyle of the humerus and the dorsal part of the olecranon. Free spots in the dorsal region of the olecranon process of the ulna (Fig. 1). Computed tomography (CT) showed a high-density macular focus and smooth edge of the lateral condyle of the right humerus and dorsal side of the ulnar olecranon. There was a high-density shadow with a clear boundary, no noticeable fracture line shadow in the elbow joint, and no noticeable swelling in the surrounding soft tissue (Fig. 2). Indicating calcification of common extensor tendinosis. Since a bony mass was noted around the lateral epicondylar region of the elbow, CT was performed to exclude the extent and characteristics of this lesion and to rule out any hostile pathology, such as malignancy. The patient had undergone conservative treatment for approximately 2 years. Her visual analog scale score before surgery was 6/10. Her pain did not respond to conservative treatment with analgesics, physiotherapy, or a rest regimen. The pain recurred more intensely 1 week prior, to the extent that she could not use her right upper limb to perform daily activities, and her sleep was severely disturbed. Therefore, surgical treatment was recommended for her. The patient then underwent surgical exploration of the elbow under general anesthesia. A right elbow lateral incision of approximately 5 cm, an incision of the subcutaneous tissue exposed to the extensor tendon, and a milky white liquid outflow from the common extensor tendon region with dystrophic calcification was made (Fig. 3). Decompression of the involved tendons and thorough cleaning were performed. Calcification was excised, and extensor tendon repair was done using 2-0 Vicryl sutures. The patient was advised to rest with minimal exercise for 1 month, followed by complete physical activities. Histopathological examination of the excised tissue revealed fibrous ligament tissue with calcifications (Fig. 4). The postoperative plain radiograph showed complete removal of the heterotopic bony growth from the lateral epicondylar area with no recurrence of the heterotopic bone formation around the elbow joint (Fig. 5). The patient had complete elimination of elbow pain (visual analog scale score 0/10), and at the follow-up of 6 months, she had no pain with full elbow function and a full range of elbow extension and flexion (Fig. 6).

## 3. Discussion

Calcific tendonitis occurs frequently in the shoulder and mainly involves a single joint. Its prevalence is highly variable (3%–22% in the general population), depending on its location within the body.^[3]^ Involvement of the elbow joint is sporadic.^[4]^ Due to the scarcity of an extensive series of such cases, the precise incidence of elbow joint involvement is unknown. Under these conditions, calcium deposits occur in the tendons, ligaments, joint capsules, and surrounding connective tissues.^[4]^ Calcific tendinitis is classified as acute, subacute, or chronic according to the extent of its clinical symptoms.^[2]^ The French Arthroscopic Society categorizes calcific tendinitis into 4 types based on the morphology of calcium deposits in radiology.^[6]^ Sandstrom divided the course of calcific tendonitis into acute, chronic, and latent forms.^[7]^ According to the Gartner and Heyer classification, this patient was classified as type 1 calcification. Acute cases are sometimes associated with pain, swelling, tenderness, restricted mobility, and fever. Calcium deposits, though seen on radiographs, may finally be absorbed spontaneously. One-third of these cases may have a history of trauma.^[8]^ The radiographic features of ossified calcification around the lateral epicondylar region may confuse these lesions with an avulsion fracture from the lateral humeral condyle, as was apparent on radiographs in this case. Chronic cases may present with less swelling and tenderness and an extended history of the illness, comparable to our case. Simple radiographs may diagnose calcific tendinitis if the calcification is substantial and matured. CT and magnetic resonance imaging may help diagnose if the plain radiographs are inconclusive. Calcific tendonitis is a cell-mediated disease wherein metaplastic transformation occurs in chondrocytes, subsequently inducing calcification. Several theories have been suggested for its etiopathogenesis, such as degeneration,^[4]^ ischemia,^[3]^ and the involvement of factors such as anti-inflammatory agents, metabolic disorders, and genetic predisposition.^[2]^ We believe that degeneration in the common extensor origin tendon was accountable for the calcification in our situation. There are several possible treatments for these conditions. These include traditional management such as nonsteroidal anti-inflammatory drugs, orally administered cimetidine,^[6]^ rest, immobilization of the affected joint, and local steroid injection. Other treatment options include needle aspiration for calcium deposits,^[7]^ shockwave therapy,^[8]^ and surgical excision. Steroidal injections may be helpful in acute cases; however, repetitive injections should be avoided, as they may lead to adverse reactions such as tendon rupture, postinjection pain, subcutaneous atrophy, and skin pigmentation. High recurrence (37%) at 1 year has been reported in patients with lateral epicondylitis who underwent local steroid injections.^[8]^ Recently, the oral route of cimetidine has been deliberated, in which calcium deposits died out in 56% of patients, reduced by 25%, and did not change in only 19%. Although cimetidine’s complete mechanism of action on periarticular calcifications remains unclear, it appears valuable in such cases. Cimetidine is supposed to act on the parathyroid hormone, boost calcium deposit absorption, and have an anti-inflammatory analgesic accomplishment.^[6]^ However, long-term studies are required to determine the side effects of drugs and treatment responses. Percutaneous aspiration is only helpful for soft calcifications.^[9]^ Shockwave treatment is a new modality but ineffective in eliminating deposits.^[10]^ Surgery is indicative of chronic and refractory disease. It may overcome the weaknesses of nonoperative treatments, as it allows complete removal with suitable sample collection for histopathological investigation and may be related to the reduced reappearance rate. We suggest surgical emptying of the calcific deposits if proven by appropriate imaging, such as a CT scan.

## 4. Conclusion

Calcific tendonitis of the elbow is not a common condition; hence, its diagnosis and treatment may be delayed due to its rarity. Therefore, appropriate imaging and treatment should be considered immediately after suspected calcified tendonitis.

## Author contributions

**Formal analysis:** Emmanuel Ackah, Zhijun Pan.

**Investigation:** Emmanuel Ackah.

**Methodology:** Emmanuel Ackah.

**Writing—original draft:** Emmanuel Ackah.

**Writing—review & editing:** Emmanuel Ackah, Deting Xue.

**Conceptualization:** Deting Xue.

**Data curation:** Deting Xue.

**Project administration:** Deting Xue.

**Resources:** Deting Xue, Zhijun Pan.

**Supervision:** Deting Xue, Zhijun Pan.

**Funding acquisition:** Zhijun Pan.
